# The dual impact of physical exercise on depression and fall risk in older Chinese adults — evidence from CHARLS 2020

**DOI:** 10.3389/fpubh.2025.1615326

**Published:** 2025-08-11

**Authors:** Wenhui Yuan, Jinghang Cui

**Affiliations:** ^1^College of Physical Education, Xi’an Shiyou University, Xi'an, China; ^2^Center for Applied Science in Health and Aging, Western Kentucky University, Bowling Green, KY, United States

**Keywords:** physical exercise, depression, falls, older adults, CHARLS 2020, urban-rural differences, mediation analysis, public health

## Abstract

**Introduction:**

Rapid population aging in China has elevated concerns regarding the mental and physical well-being of older adults. This study investigates the interrelationships among physical exercise, depression, and fall risk using data from the 2020 wave of the China Health and Retirement Longitudinal Study (CHARLS).

**Methods:**

We analyzed a sample of 3,694 older adults. An ordinary least squares regression model was employed to assess the impact of physical exercise on depression, while a logistic regression model was used to examine the effect on fall risk. Key control variables included age, biological sex, income, marital status, and major accident history. Mediation analyses were then conducted to test the indirect effect of exercise on fall risk through depression, with subgroup analyses comparing urban and rural respondents.

**Results:**

Results from the depression model revealed that physical exercise significantly reduced depression scores ( β=−0.3156; p=0.002 ), and males exhibited lower scores relative to females. The falls model indicated a trend toward reduced fall risk with increased exercise ( β=−0.0738;OR≈0.929;p=0.061 ), while older age and female gender are associated with elevated risk. Mediation analysis demonstrated a significant indirect effect in the urban group (mean indirect effect = −0.0338), but not in the rural group (mean indirect effect = −0.0056).

**Discussion:**

These findings suggest that physical exercise not only directly improves mental health but also indirectly reduces fall risk through alleviating depressive symptoms in urban older adults. The lack of a significant mediating effect in rural areas suggests that local contextual factors may alter the exercise-depression-fall pathway. The results support integrated public health interventions tailored to local settings to enhance both mental and physical outcomes among China’s aging population.

## 1 Introduction

Falls and depression are two major threats to healthy aging. Falls are the leading cause of injury and disability in older adults worldwide, with roughly one in three adults over 65 experiencing at least one fall each year (1). The consequences of falls range from fractures and loss of independence to increased mortality and healthcare costs (2, 3). At the same time, depression is highly prevalent in late life: globally over 320 million people suffer from depression, representing a significant cause of disability (4). China has the world’s largest older population, and late-life depression has become a serious public health concern in the Chinese context (5, 6). Both falls and depression not only impair quality of life for older individuals but also impose heavy burdens on families and society.

Growing evidence suggests that an active lifestyle can confer protective benefits against both depression and falls in older adults. Regular physical activity is well known to support mental health, Schuch et al. (7) found that people with higher physical activity levels had about 17% lower odds of developing depression compared to those with low activity. A substantial body of literature indicates that regular physical activity can play a crucial role in promoting mental health (8). Physical exercise has been shown to reduce depressive symptoms and improve mood among older adults (9). Meta-analyses of randomized controlled trials have reported large antidepressant effects associated with exercise, while longitudinal research suggests that meeting physical activity guidelines can lower the odds of developing depression by as much as 23% (10). These findings underline the theoretical perspective that physical activity enhances psychological well-being by triggering neurobiological, psychosocial, and behavioral mechanisms. In older adult populations, even moderate exercise has been associated with reduced depressive symptoms. A recent study of Chinese adults aged over 60 reported that engaging in high-intensity physical exercise was linked to significantly lower depression scores (9). Conversely, sedentary or inactive lifestyles may heighten depression risk (11).

Physical activity also plays a key role in fall prevention. Maintaining strength, balance, and mobility through exercise is one of the most effective strategies to reduce falls in older age (12). Numerous intervention trials have shown that exercise programs can prevent falls by improving physical function. In contrast, low levels of activity are a known risk factor for falling (13). Thus, physical inactivity may contribute to both greater depression and higher fall susceptibility in the older adult. Depression itself can independently increase the risk of falls. Physiological changes associated with depressive disorders, such as gait impairment, slowed reaction, poorer concentration, and side effects of antidepressant medications – may predispose older adults to falling. Epidemiological studies consistently find that seniors with depressive symptoms are more likely to experience falls. A meta-analysis of 20 prospective studies concluded that late-life depression is a significant risk factor for falls, regardless of how depression or falls were measured (14). In China, recent nationwide data have reinforced this link: for example, an analysis of the 2018 China Health and Retirement Longitudinal Study (CHARLS) found that older adults with depressive symptoms had 1.37 times higher odds of falling compared to those without depression (2). These findings underscore that depression can exacerbate fall risk, making it important to consider mental health in fall prevention efforts.

Physical exercise may reduce fall-risk in older adults not only through direct improvements in muscle strength and balance but also indirectly by alleviating depressive symptoms. Biologically, regular exercise increases neurotrophic factors, enhances neurotransmitter regulation, and reduces systemic inflammation, all of which contributes to improved mood and cognitive function (15). Psychologically, exercise fosters self-efficacy and positive affect, counteracting hopelessness and psychomotor slowing characteristic of depression (16). Socially, group-based activities provide social support and reduce isolation--factors known to buffer against depressive symptomatology (17). In turn, lower levels of depression lead to better attention, faster reaction times, and greater willingness to engage in balance-challenging activities, thereby reducing the likelihood of slips and falls (18). Together, these biological, psychological, and social pathways form the rationale for testing depression as a mediator of the exercise to falls relationship. Therefore, this paper puts forward research hypothesis 1 and hypothesis 2:

*H1*: Other things being equal, higher physical exercise levels associated with lower depressive symptom severity and lower odds of falls in older adults.

*H2*: Other things being equal, depressive symptoms mediate the relationship between physical activity and falls.

Another critical dimension in these interrelationships is the influence of environmental context – in particular, urban versus rural residence. Emerging research from China has highlighted that urban–rural disparities significantly shape health outcomes in later life. Urban residents typically have greater access to recreational facilities, structured exercise programs, and healthcare services, which may enhance the beneficial effects of physical activity on both mental and physical health. Prior studies indicate substantial urban–rural disparities in physical activity, depression, and falls among Chinese older adults. Generally, rural elders tend to have lower socioeconomic status and poorer access to health resources than urban elders, which may translate into different health outcomes. National survey data show that late-life depression is markedly more common in rural areas (approximately 44% prevalence) than in urban areas (about 32%) (19). Likewise, the incidence of falls among older adults in rural China has been reported to exceed that of urban seniors. Differences in lifestyle may play a role: rural residents often engage in more occupational or household physical labor yet have less access to organized exercise activities (20). Urban older adults, on the other hand, might have more opportunities for leisure-time exercise but also more sedentary behavior. These contextual differences can modify how physical activity and depression impact fall risk. For instance, one CHARLS-based study observed that the association between depressive symptoms and falls was significant in rural older adult but not in urban older adult, possibly due to better healthcare and living conditions in cities (21).

Moreover, the strength of the association between depression and fall risk might also vary by residential context, with some evidence indicating a more pronounced impact in rural settings (22). A recent analysis found that higher-intensity exercise was associated with less depression among urban older adults, whereas rural elders showed generally low activity levels and higher depression, attenuating the correlation in rural areas (9). These findings suggest that urban–rural context can moderate the links among physical activity, depression, and falls, warranting explicit investigation (23). Existing literature indicates that staying physically active can improve mood and reduce fall risk in older adults, and that depression can in turn elevate fall risk. We explicitly consider depression as a potential mediator between physical activity and falls, and test whether urban–rural residence moderates these associations. Therefore, this paper puts forward research hypothesis 3:

*H3*: The mediating effect differ between urban and rural older adults.

Most prior studies have examined these factors in pairs, focusing on exercise and depression, or depression and falls – rather than addressing the three variables together. There is a paucity of research examining the interconnected pathways by which physical activity may influence falls via depression in older populations, especially in China. Few studies have explored whether these interrelationships operate differently in rural versus urban communities. To fill these gaps, the present study uses data from the nationally representative CHARLS 2020 wave to investigate the interconnections among physical activity, depression, and fall risk in Chinese adults aged 60 and above. By simultaneously addressing physical activity, mental health, and fall outcomes in a single framework, this study offers a novel comprehensive perspective. Clarifying these interconnections in a large, nationally representative sample of Chinese older adults can inform more integrated intervention strategies tailored to different community settings.

## 2 Materials and methods

### 2.1 Samples and data sources

The data utilized in this study were derived from the China Health and Retirement Longitudinal Study (24). CHARLS is a nationally representative, longitudinal survey initiated and conducted by the National Development Research Institute at Peking University. The program targets community-dwelling Chinese residents aged 45 years and older, as well as their spouses, collecting comprehensive, multidimensional information concerning socioeconomic status, health conditions, and family dynamics. The survey adopts probability sampling proportional to population size, encompassing 450 communities or villages within 150 counties and districts across China, ensuring extensive geographic coverage and representativeness (25). Ethical approval for all survey rounds was provided by the Biomedical Ethics Committee of Peking University, and informed written consent was obtained from all participants prior to data collection. Survey data and detailed information about the CHARLS can be accessed through its official website.^1^

**Figure 1:**
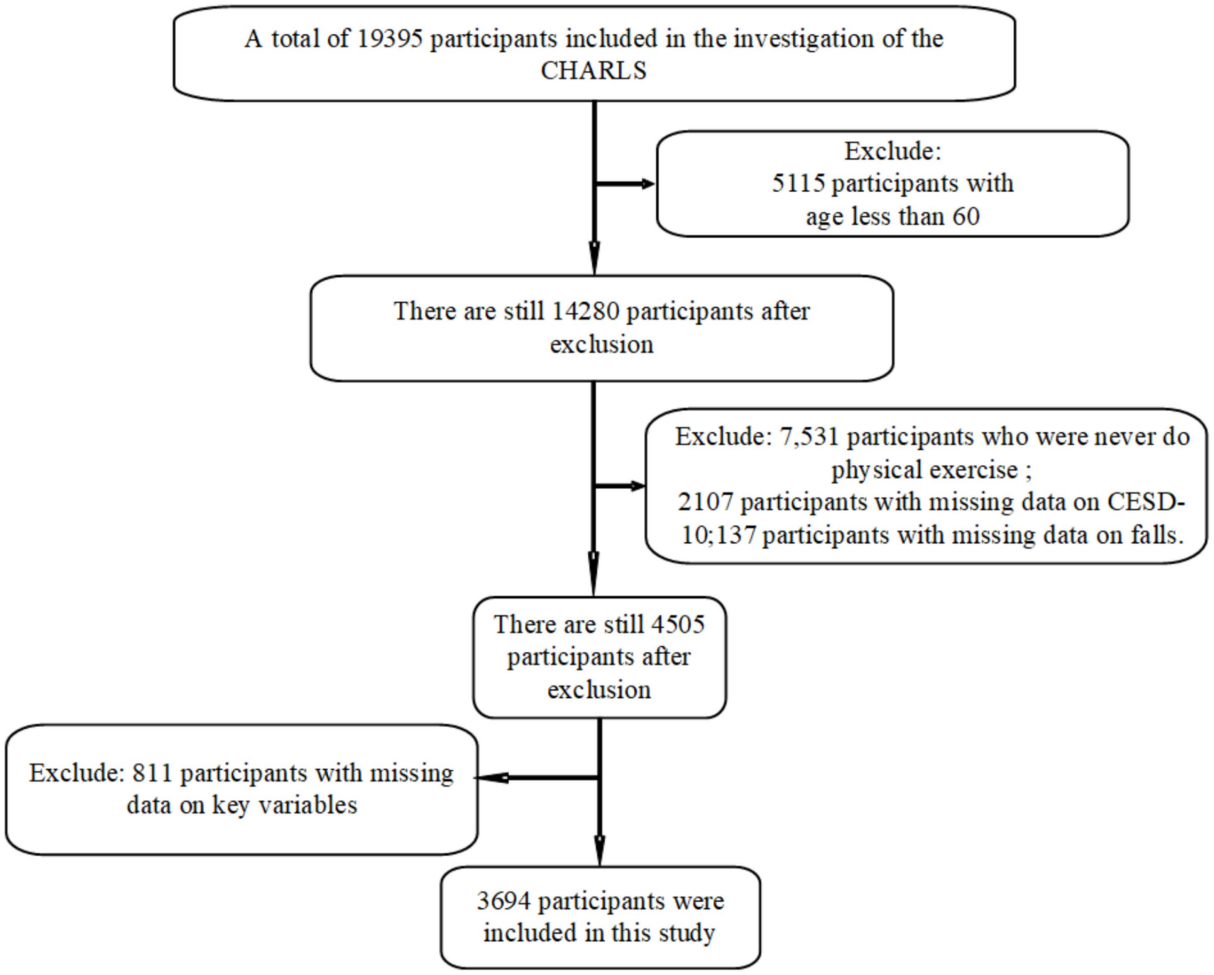
Flow chart of the selection process of participants.

For our analysis, we used the 2020 wave of CHARLS data, which included a total of 19,395 subjects. Among them, respondents who are younger than 60 years old, or did not provide consent or had incomplete gender information, or no physical exercise, falls, and depression status information at all were excluded from subsequent analyses. Finally, we included 3,694 respondents in the final study. The detailed exclusion process is shown in Figure 1.

### 2.2 Physical exercise assessment

Physical activity was assessed via the standard CHARLS questionnaire, which collects self-reported information on the frequency and intensity of exercise or physical labor. Participants were asked how often they engage in moderate or vigorous physical activities (such as heavy household work, running, calisthenics, etc.) in a typical week. Following the CHARLS coding, we combined frequency and intensity responses to categorize each individual’s overall physical activity level on a 5-point ordinal scale (26). Level 1 corresponded to being essentially inactive (sedentary), reporting little to no moderate or vigorous activity in a usual week. Level 2 indicated low activity – some light or moderate physical activity but less than the recommended frequency. Level 3 represented moderate activity (engaging in moderate exercise a few days per week, or light activities daily). Level 4 denoted high activity – regular exercise most days, including some vigorous-intensity workouts. Level 5 corresponded to very high activity levels (frequent vigorous exercise or physically demanding labor on a daily basis).

The 5-level exercise variable is derived directly from CHARLS codebook categories. Prior CHARLS validation studies have demonstrated that these self-reported intensity levels correlate strongly with objective accelerometer measures (27) and approximate the WHO physical activity guidelines of ≥150 min/week of moderate-intensity or ≥75 min/week of vigorous-intensity activity when levels ≥2 are combined (28).

### 2.3 Depressive symptoms assessment

Depression was measured using the short form of the Center for Epidemiologic Studies Depression Scale (CES-D 10) included in CHARLS. Respondents were asked ten questions about how often in the past week they experienced various symptoms, such as feeling depressed, lonely, or having trouble sleeping. Each item is rated on a 4-point Likert scale for frequency. In CHARLS, these items were scored from 1 to 4 and summed to create a total depression score. Thus, the composite CES-D score ranges from 10 (indicating no depressive symptoms on any item) to 40 (severe symptoms on all items) (29). Higher scores correspond to greater depressive symptom severity (30).

### 2.4 Falls assessment

The occurrence of falls was assessed with a binary self-report item. CHARLS asked each respondent: “Have you fallen down in the last 2 years (since the last survey)?.” Falls were defined for participants as unintentionally coming to rest on the ground or floor (excluding major accidents). We coded the falls variable as 1 if the participant reported one or more falls in the past 2 years, and 0 if they reported no falls. Thus, “fall risk” in our study refers to the likelihood of having had at least one fall within the recent two-year period (2).

### 2.5 Covariates

We controlled for a range of demographic and health covariates known to be associated with physical activity, depression, or falls in older adults. These covariates (all measured in the CHARLS 2020 survey) included: Age, treated as a continuous variable or categorized into age bands (60–69, 70–79, ≥80) for some analyses; Biological sex (male or female); Income (gross annual income level); Marital status (Never married, married, divorced, widowed); Accident (ever experienced a major accidental injury or not), which we treat as a continuous covariate. Finally, we include residential region (urban vs. rural locality) as a key moderating variable. Residential region was classified according to the CHARLS community administrative codes (21). In this system, communities are first assigned an administrative “region type” by local government: Urban: districts and sub-districts of prefecture-level cities, indicating built-up areas with municipal infrastructure; Rural: counties, townships, and villages, indicating predominantly agricultural or nonurbanized areas. We collapsed these into a binary variable (urban vs. rural) for our analyses. All covariates were chosen *a priori* based on the literature and prior CHARLS studies linking these factors to depression or fall outcomes (31, 32). By adjusting for these characteristics, we aimed to account for potential confounding influences and isolate the relationships of interest among physical activity, depression, and falls.

### 2.6 Statistical analysis

An ordinary least squares (OLS) linear regression was used to examine the association between physical exercise levels and depressive symptom scores. In parallel, a logistic regression model was implemented to assess the association between physical exercise and the probability of falls. These analyses allowed the estimation of regression coefficients for continuous outcomes and adjusted OR for binary outcomes, effectively quantifying the direct impact of physical inactivity on both depression and fall risk.

The mediation analysis sought to determine whether depressive symptoms serve as an intermediary mechanism linking physical exercise to fall risk. In this framework, physical exercise was hypothesized to influence depressive symptoms (path a), which in turn affects fall risk (path b). The study quantified the indirect effect by calculating the product of the coefficients derived from the physical exercise → depression → falls pathways. To robustly assess the significance of this indirect effect, a nonparametric bootstrapping approach with 5,000 resamples was adopted, yielding a bias-corrected 95% confidence interval. In addition, the analysis addressed potential contextual influences by testing for moderation effects of urban versus rural residence. Interaction terms were incorporated in both the depression and falls models to evaluate whether the strength of the associations differed by residence. Stratified analyses were conducted separately for urban and rural subgroups to compare the magnitude of the indirect effects of exercise on falls via depressive symptoms. An index of moderated mediation was also computed and its confidence interval derived from bootstrap samples to formally test for differences in mediation effects across the subgroups.

All statistical analyses were performed using Python version 3.6.4. The level of significance was set at *p*-values < 0.05.

## 3 Results

### 3.1 Descriptive statistics

Table 1 summarizes the main characteristics of the study sample ( N=3,694 ). The variable “Biological sex” was self-reported in CHARLS, with participants indicating either male or female. With urban residence coded as 1 and rural as 0, the average value suggests that approximately 44% of the respondents live in urban areas, whereas 56% reside in rural regions. Falls were captured as a binary outcome: 666 participants (18.0%) reported having experienced a fall, while 3,028 (82.0%) did not. Marital status distribution was 185 (5.0%) never married, 2,242 (60.7%) married, 370 (10.0%) divorced, and 897 (24.3%) widowed. Income category (gross annual income) was grouped as follows: ≤2000¥(513;13.9%) , 2001–5000¥(1322;35.8%) , 5001–20000¥(1064;28.8%) , 20001–50000¥(624;16.9%) , and >50000¥(171;4.6%).

**Table 1:** Descriptive statistics.

Variables	Category/Description	*N*	%/Mean ± SD	VIF
Biological sex	Male	1847	50.00%	1.8
	Female	1847	50.00%	
Residential region	Urban	1,626	44.00%	2.3
	Rural	2068	56.00%	
Fall	Yes	3,028	82.00%	2.1
	No	666	18.00%	
Marital status	Never married	185	5.00%	3.1
	Married	2,242	60.70%	
	Divorced	370	10.00%	
	Widowed	897	24.30%	
Income category	≤ 2000 ¥	513	13.90%	2.8
	2001–5,000 ¥	1,322	35.80%	
	5,001–20,000 ¥	1,064	28.80%	
	20,001–50,000 ¥	624	16.90%	
	> 50,000 ¥	171	4.60%	
Exercise	Level: 1–5	3,694	3.03 ± 1.10	1.9
Age (years)	/	3,694	68.4 ± 6.1	2.2
Depression score (CES-D10, 10–40)	/	3,694	22.0 ± 7.0	2.4

For continuous measures, we report mean±SD : Physical exercise level (1 = lowest to 5 = highest) averaged 3.03±1.10 , indicating a moderate level of physical activity across the sample. Age was 68.4±6.1 years, reflecting a sample primarily in the late 60s. Depression score (CES-D10, range 10–40) averaged 22.0±7.0 , suggesting moderate depressive symptoms. This balanced distribution of sex and region, together with moderate levels of exercise and depression and an 18% fall prevalence, provides a solid basis for examining how physical activity, mood, and fall risk interrelate in older Chinese adults. And as shown in Table 1 that every variables’ VIF is below 4, indicating that multicollinearity is unlikely to distort our results.

### 3.2 Effects of physical exercise

#### 3.2.1 Impact on depression

The OLS regression was estimated with the Depression Score as the dependent variable ( N=3,694 ). The model explained approximately 4.5% of the variation in depression (*R*^2^ = 0.045), and the overall model was statistically significant. The key results are shown in Table 2.

**Table 2:** OLS model for depression.

Variables	Coefficient	SE	*z*-value	*p*-value	95% Confidence interval
Exercise	−0.316	0.103	−3.070	0.002	[−0.518, −0.114]
Age	0.018	0.019	0.914	0.361	[−0.020, 0.056]
biological sex	−2.040	0.232	−8.790	0.000	[−2.490, −1.590]
Income	−0.334	0.069	−4.820	0.000	[−0.470, −0.198]
Marital status	0.790	0.149	5.280	0.000	[0.496, 1.082]
Accident	−0.074	0.673	−0.110	0.913	[−1.392, 1.245]

Holding age, biological sex, income, and marital status constant, each additional unit increase in the physical exercise index is associated with an average decrease of 0.315 points in the Depression Score. This significant negative effect supports H1, that increased physical exercise is related to improved (i.e., lower) depression levels. The coefficient for age is not statistically significant, suggesting that after accounting for other factors, age does not contribute meaningfully to the variation in depression scores. The negative coefficient implies that on average, males have lower depression scores than females of about 2.04 points. In other words, older men tend to have lower depression scores relative to older women, a difference that is highly significant. Higher income is associated with lower depression scores, suggesting that better economic conditions have a protective effect on mental health. The positive coefficient indicates that as the marital status code increases (from never married to widowed), the Depression Score tends to increase. This finding suggests that, compared to never married individuals, those who are married, divorced, or widowed have higher depression scores. This could reflect the differential social and emotional challenges associated with these marital states in later life. The history of major accidents does not significantly predict depression scores when other variables are controlled, indicating that, in this sample, accident history has minimal impact on depressive symptoms.

The OLS model demonstrates that physical exercise is significantly associated with reduced depressive symptoms among older adults—supporting the H1 hypothesis. Additionally, biological sex differences are pronounced; with males showing lower depression than females, which may reflect both behavioral and psychosocial differences. The positive effect of higher marital status codes suggests that those with more complex marital histories (particularly divorced or widowed individuals) may be at increased risk for depression, while higher income appears protective.

#### 3.2.2 Impact on falls

A logistic regression was performed with Fall as the outcome, using the same set of predictors. Although the overall model explained a modest amount of variance (Pseudo *R*^2^ = 0.01925), the likelihood ratio test was significant, indicating that at least one predictor is significantly associated with fall risk. The key results are shown in Table 3.

**Table 3:** Logistic model for fall risk.

Variables	Coefficient	SE	*z*-value	*p*-value	95% Confidence Interval	OR
Exercise	−0.074	0.039	−1.870	0.041	[−0.151, 0.003]	0.929
Age	0.020	0.007	2.660	0.008	[0.005, 0.034]	1.020
Biological sex	−0.583	0.091	−6.400	0.010	[−0.760, −0.403]	0.558
Income	−0.011	0.027	−0.420	0.674	[−0.064, 0.041]	0.989
Marital status	0.100	0.054	1.860	0.063	[−0.006, 0.206]	1.105
Accident	−0.105	0.265	−0.395	0.693	[−0.623, 0.414]	0.901

The coefficient for physical exercise is negative, suggesting that higher levels of exercise are related to a lower risk of falls. The corresponding OR implies that each unit increase in exercise reduces the odds of falling by about 7.1%. Although this effect is in the hypothesized direction, its *p*-value of approximately 0.041 indicates that the result is marginally significant. The odds ratio for exercise on falls (OR = 0.93) suggests a trend toward reduced fall risk with higher activity, though the effect size is modest and marginally significant. A significant positive coefficient confirms that each additional year of age increases the risk of falling. This finding aligns with expectations that aging is associated with higher fall risk, likely due to physiological decline. With biological sex coded as female = 0 and male = 1, the negative coefficient suggests that males have lower log odds of falling compared to females. The OR indicates that males’ odds of falling are roughly 56% those of females, meaning that female older adults face a significantly higher risk of falling. The coefficient for income is not statistically significant in the fall model, indicating that economic factors do not have a clear association with fall risk within this sample. The effect of marital status on fall risk is marginally significant, with a positive coefficient suggesting a potential increase in fall risk as the marital status code increases. Although this effect is less robust than those of other variables, it implies that individuals with more complex marital experiences (e.g., divorced or widowed) might be at slightly higher risk for falls. Major accident history does not significantly predict falls, implying no substantial influence on fall risk after adjusting for other factors.

The logistic regression results indicate a marginally significant protective effect of physical exercise on falls—supporting the hypothesis 1 that exercise is beneficial, although the statistical evidence for this association is not as strong as that observed for depression. Furthermore, significant effects for age and biological sex highlight that older age increases fall risk, while females are at a higher risk of falling compared to males. The role of marital status, while only marginally significant, suggests that the social and emotional challenges associated with marital disruption may contribute to an increased fall risk.

Taken together, the results from the depression and fall models provide partial support for the overall hypothesis 1—that physical exercise is significantly associated with both lower depressive symptoms and, to some extent, reduced fall risk in older adults. The findings support the notion that physical exercise is beneficial for older adults by contributing to both improved mental health and reduced physical risk. The differential effects by biological sex and the influence of marital status offer important contextual insights. Women and those with less favorable marital experiences are particularly vulnerable to higher depression levels, which in turn may compound their risk of falling. These outcomes underscore the importance of incorporating targeted interventions that promote physical exercise and address psychosocial support among high-risk subgroups, ultimately contributing to healthier and safer aging.

### 3.3 Mediation analysis

**Figure 2:**
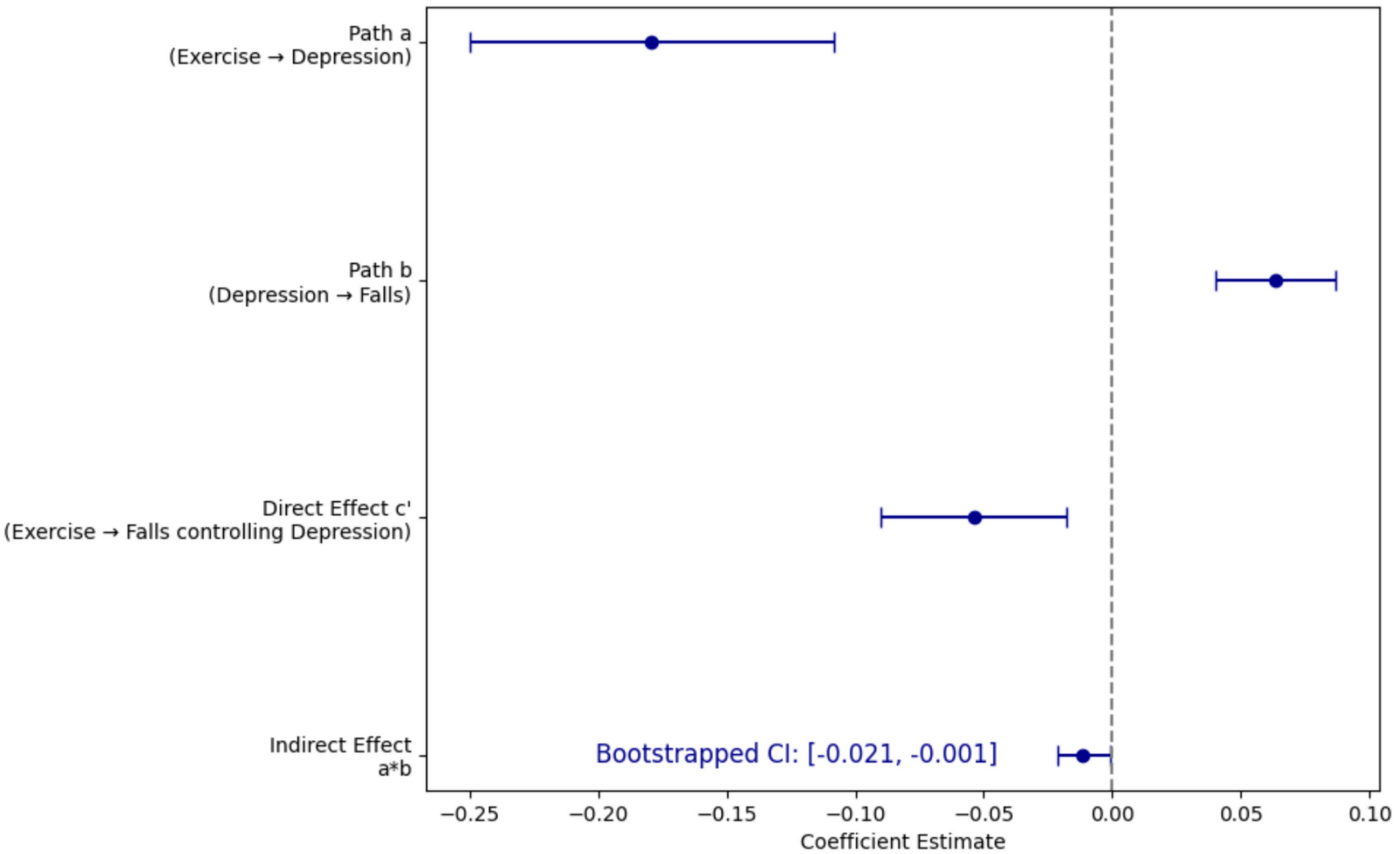
Forest plot of mediation paths.

To explore whether depression mediates the relationship between physical exercise and fall risk, a mediation analysis was performed incorporating both the OLS model for depression and the logistic regression model for falls. The results are shown in Figure 2 and summarized as follows.

Path a (Exercise → Depression): The coefficient was −0.1792 ( p=0.017 ), showing that increases in physical exercise were significantly associated with lower depression scores; Path b (Depression → Falls): In the logistic regression controlling for physical exercise, each one-unit increase in depression was associated with an increase in the log-odds of falling by approximately 0.0633. Indirect Effect (a*b): The computed indirect effect was −0.01135. Bootstrapping (5,000 samples) yielded a 95% confidence interval of [−0.021, −0.001]. Because the confidence interval did not include 0, the mediation effect is significant, indicating that part of the protective effect of exercise on falls is mediated through its effect on reducing depression. Direct Effect (Path c′): The direct effect of physical exercise on falls, after accounting for depression, was −0.05386, suggesting that exercise also has an independent effect on reducing fall risk aside from its mediated pathway through depression.

**Figure 3:**
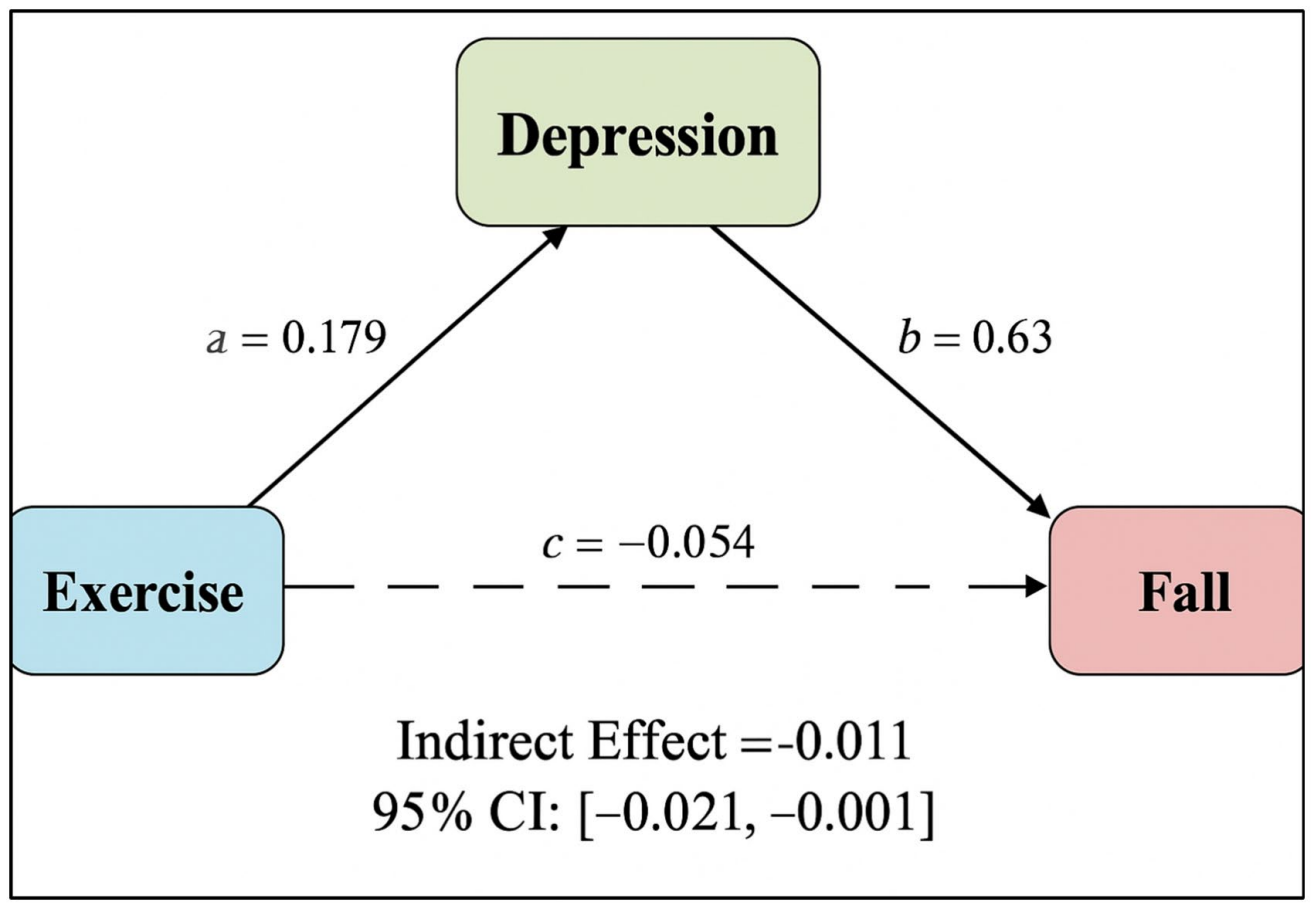
Diagram of the mediation effect.

The mediation analysis supports H2, which posits that depression mediates the relationship between physical exercise and fall risk. Specifically, the significant negative coefficient in Path a indicates that more exercise is linked to lower levels of depression. Concurrently, the significant positive coefficient in Path b confirms that increased depression is associated with a higher risk of falling. The significant indirect effect demonstrates that exercise indirectly reduces fall risk by mitigating depressive symptoms. Additionally, the presence of a significant direct effect indicates that exercise not only benefits fall risk via a mental health pathway but also exerts an independent protective influence, suggesting partial mediation. These findings indicate that encouraging physical exercise among older adults may help reduce fall risk both by directly enhancing physical function and by improving mental health. This dual pathway has important implications for interventions aiming to promote safety and quality of life in aging populations. The diagram of the mediation effect is shown in Figure 3.

### 3.4 Subgroup analysis: urban–rural differences

**Figure 4:**
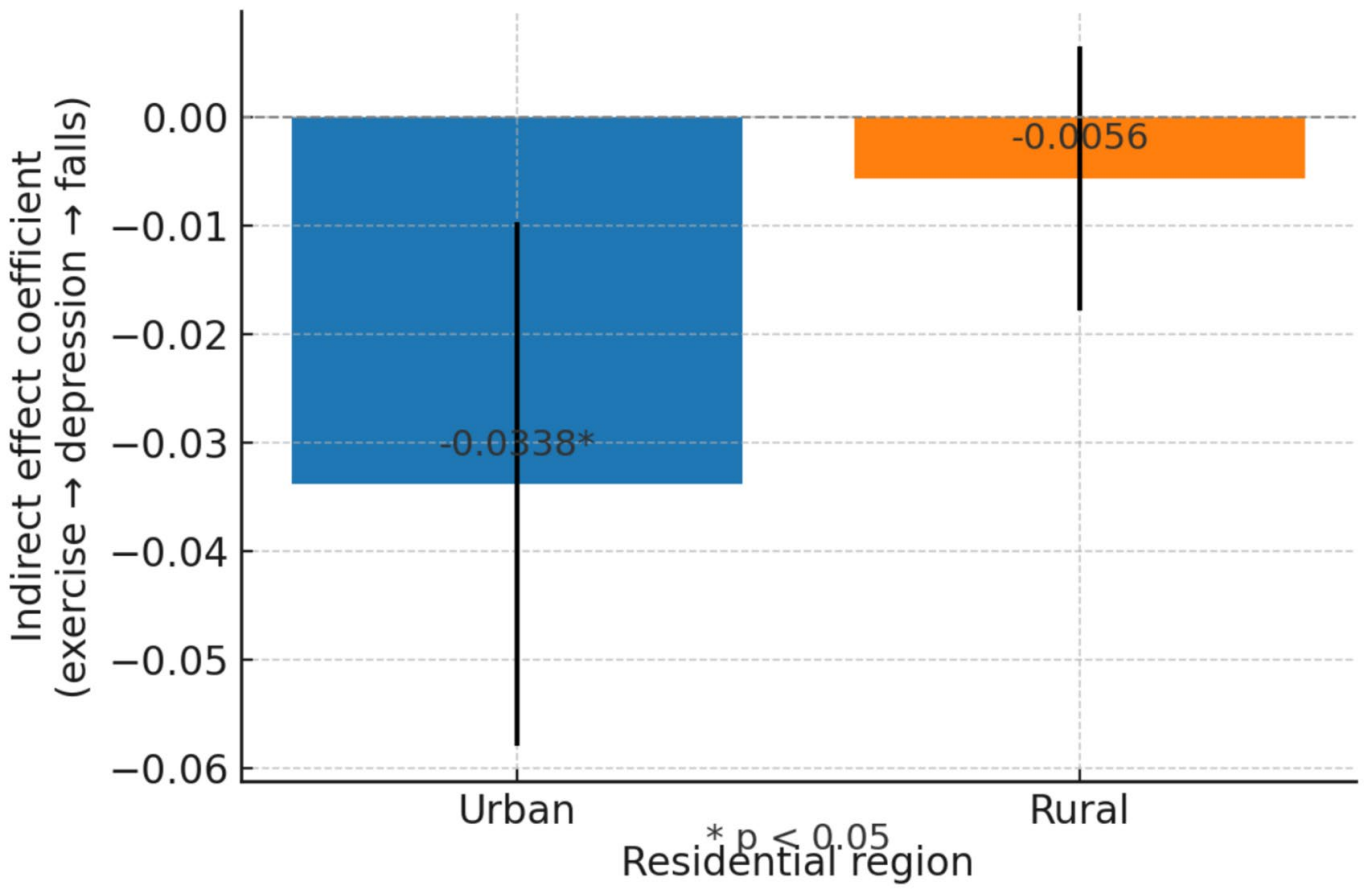
Conditional indirect effects by residence.

Given the contextual variations in health outcomes across urban and rural settings, subgroup analyses were conducted to test for the moderating role of urban versus rural residency on the exercise–depression–fall pathway. The mediation effect was evaluated separately for urban and rural groups. The conditional indirect effects by residence are shown in Figure 4.

#### 3.4.1 Urban group

The 95% confidence interval does not include 0, indicating that the indirect effect of physical exercise on fall risk—operating through reduced depression—is statistically significant in the urban subgroup. The negative value of the mean indirect effect confirms that an increase in physical exercise is associated with a reduction in depression, and that lower depression, in turn, is linked to lower fall risk. The overall result is a significant reduction in fall risk mediated by improved mental health. This finding suggests that among urban older adults, exercise not only has a direct protective effect but also contributes to fall prevention by alleviating depressive symptoms. Urban dwellers may benefit from structured exercise opportunities and social settings that amplify the positive impact of physical activity on mental health.

#### 3.4.2 Rural group

In contrast, for rural older adults, the confidence interval includes 0, which indicates that the mediated effect of physical exercise on fall risk via depression is not statistically significant. Although the direction of the indirect effect is negative, it’s very small magnitude and lack of significance suggest that the mechanism through which exercise reduces depression—and in turn lowers fall risk—is not as pronounced in the rural subgroup. This may imply that among rural older adults, either the benefits of exercise in reducing depression are attenuated, or that other factors (e.g., baseline physical workload, limited access to organized exercise programs, or different social support structures) play a more dominant role in influencing fall risk.

The subgroup analyses indicate that the indirect effect of physical exercise on fall risk via depression is significantly stronger in urban older adults than in their rural counterparts. These results provide empirical support for H3, suggesting that the residential environment moderates the mediation pathway.

In urban environments, older adults likely have greater access to organized exercise programs, better community resources, and more opportunities for social interaction, which may enhance the mood-improving effects of physical activity and thereby reduce the risk of falls. In contrast, rural older adults might experience other intervening factors, such as occupational physical activity, less structured forms of exercise, or different psychosocial stressors, which dilute the mediated pathway from exercise through depression to falls. The observed difference underscores the need for context-specific interventions. Urban initiatives might effectively leverage group-based exercises to address both mental health and physical safety, whereas rural interventions may require additional components to bolster their impact on depression and fall prevention.

The mediation analysis reveals a clear and significant indirect effect of physical exercise on fall risk via reduced depression among urban older adults, while in the rural subgroup, this indirect pathway is not significant. These results substantiate H3 by demonstrating that the environment plays a crucial role in moderating how exercise impacts fall risk through mental health improvements, emphasizing that tailored strategies are needed for different residential settings.

### 3.5 Robustness checks

To confirm the stability of our primary results, we performed several robustness checks using sampling weights model and goodness-of-fit tests. Given that CHARLS employs a complex sampling design, we conducted sensitivity analyses using sampling weights to yield estimates more representative of the target population. The weighted regression models, for both OLS and logistic specifications, produced similar point estimates and significance levels relative to the unweighted models. This further reinforces the reliability of our findings, suggesting that the sampling design does not materially alter the observed associations. The results are shown in Table 4.

**Table 4:** Robustness check results using sampling weights model.

Model Type	Specification	Coefficient	SE	*p*-value	Additional metric
OLS (Depression Score)	Base model	−0.315	0.103	0.002	*R*^2^ = 0.045, Adj. *R*^2^ = 0.043
	Weighted regression	−0.317	0.102	0.002	*R*^2^ ≈ 0.045
Logistic (Fall)	Base model	−0.074	0.039	0.062	OR = 0.929; Pseudo *R*^2^ = 0.019
	Weighted regression	−0.074	0.039	0.061	OR = 0.929

To further assess the adequacy of the model, we conducted a Hosmer–Lemeshow goodness-of-fit test. The results are shown in Table 5. The Hosmer–Lemeshow test yielded a chi-square statistic of 4.220 with 8 degrees of freedom, and an associated *p*-value of 0.837. A p-value well above the conventional cutoff indicates that there is no significant discrepancy between the observed and expected frequencies of falls across deciles of predicted probabilities. In other words, the logistic model exhibits acceptable fit and the assumptions are not violated. The favorable Hosmer–Lemeshow test result supports the robustness and stability of our logistic regression model. This suggests that our findings regarding the relationship between physical exercise and fall risk are reliable and not driven by model misspecification.

**Table 5:** Robustness check results for the logistic regression model (Fall).

Statistic	Value
Pseudo R^2^	0.020
Log-Likelihood (Model)	−1701.3
LLR *p*-value	3.65e^−12^
Hosmer–Lemeshow Chi-Square	4.220
Degrees of Freedom	8
Hosmer–Lemeshow *p*-value	0.837

These robustness checks demonstrate that the protective effects of increased physical exercise on reducing depression and fall risk are stable across multiple model specifications and weighting procedures.

## 4 Discussion

The results of this study imply that lower depressive symptoms may partly account for the association between exercise and falls, which is consistent with our theoretical model. Earlier cross-sectional data from CHARLS 2018 showed that having depression was associated with a higher likelihood of falling (21). Longitudinal analyses reinforce this relationship: in one 3-year cohort study, older adults with high depressive symptom scores at baseline had 34% higher odds of experiencing a fall during follow-up (33). Stubbs et al. (34) found that older adults diagnosed with major depressive disorder had roughly four times the odds of falling compared to non-depressed elders. The contribution of our work is in demonstrating this relationship in a contemporary Chinese cohort and showing that part of exercise’s protective effect against falls operates through reducing depression.

Several mechanisms may explain why physical exercise reduces both depression and falls in older adults. Biologically, physical activity can lead to positive neurochemical changes – including the release of endorphins and growth factors – which promote neural plasticity and improved mood regulation (35). Psychologically, exercise often enhances self-esteem and self-efficacy and provides a sense of accomplishment (36). In older adults, many forms of exercise (such as dancing, tai chi, or group exercises) are also social activities; they facilitate social interaction and reduce loneliness (37). In the Chinese context, for example, it is common for urban seniors to gather in parks for group exercise, which not only improves fitness but also fosters social support. This social engagement can alleviate feelings of isolation and produce emotional comfort, thereby reducing depressive symptoms (38). Prior studies have noted that exercising together with family or community members can strengthen intergenerational bonds and emotional wellbeing in older Chinese (39). These psychosocial benefits help explain our finding that those who exercise more tend to have better mental health. Physical exercise also directly improves physiological capacities that are crucial for preventing falls. Thus, it appears that physical activity confers a dual benefit: it uplifts mood and it bolsters physical functioning and both of which contribute to lowering an older person’s risk of falls.

Depression itself likely influences fall risk through several pathways, which helps make sense of its mediating role (40). From a physical standpoint, depression in the older adult is often accompanied by psychomotor slowing, fatigue, and loss of coordination; older adults with depressive symptoms sometimes exhibit slower walking speed and poorer balance, possibly due to diminished energy or medication side effects (41). Cognitive impairments associated with depression – such as impaired attention, executive dysfunction, and slower information processing – may also increase fall risk. Social isolation, which is both a contributor to and a consequence of depression, has also been linked to higher fall risk (42). Alleviating depression through exercise can therefore break some of these vicious cycles. For example, improving mood might increase an individual’s motivation to stay active and socially engaged, which further protects against falls (43).

An interesting finding in our study is that the mediation effect of depression on the relationship between exercise and falls appeared to differ between urban and rural residents. Specifically, the indirect effect of exercise (through reducing depression) was somewhat more pronounced among urban older adults than among rural older adults (as indicated by a stronger mediation pathway in the urban subsample). One possible explanation is the differing lifestyle and support contexts in urban vs. rural areas (44). Urban seniors may have more opportunities for structured exercise (fitness groups, community centers) and are more likely to exercise for recreation, whereas rural elders might get physical activity from labor or daily chores rather than intentional exercise. The type of exercise urban older adults engages in (tai chi, dancing, etc.) often has a social component, which could amplify its impact on depression (45). Thus, when an urban elder becomes more physically active, they might experience a notable improvement in mood due to both the exercise itself and increased social interaction, leading to a substantial mediation effect on fall risk (46). Rural older adults, by contrast, tend to have higher baseline levels of depression and fewer exercise resources. Their depression may stem from factors like isolation (e.g., “left-behind” older adults whose children have migrated) or chronic health issues that exercise alone cannot fully address (47). In our data, exercise was still beneficial in rural areas, but its effect on depression (and thereby on falls) was not as strong as in cities. It is also possible that some physical activities common in rural settings (e.g., farm work) improve strength but do less for social engagement, yielding a relatively smaller mood benefit (48). Future research should explore these urban–rural differences in more depth.

Our findings carry implications for designing interventions and policies to improve older adult health, especially in China’s context of a large aging population with diverse urban and rural settings. Firstly, the results highlight physical exercise as a key modifiable behavior that can yield concurrent benefits for mental well-being and fall prevention. This suggests that public health programs aiming to reduce late-life depression or to prevent falls should consider incorporating a physical activity component (14). For instance, community-based exercise programs could be implemented as integrated interventions to address both depression and falls risk among the older adult. Such programs would effectively “kill two birds with one stone,” improving quality of life on multiple fronts (49). In urban areas, policy makers can capitalize on existing infrastructure like parks, senior centers, and recreational facilities to organize regular exercise and social activities for seniors. Ensuring these activities are accessible and culturally appealing can encourage participation (50).

In rural areas, where formal exercise facilities are fewer, interventions might leverage community spaces or integrate physical activity into daily routines. For example, local health workers could organize morning group exercises or home-based strength training using affordable equipment. Since rural elders often have higher depression rates and face healthcare access barriers, bringing exercise programs to villages could serve as an accessible mental health promotion strategy (51). Our study’s evidence that exercise benefits mental health is particularly relevant to China’s ongoing efforts to improve depression screening and treatment in primary care. It reinforces the idea that holistic interventions are needed: not only treating depression with medication or therapy, but also prescribing lifestyle changes like increased physical activity as part of routine geriatric care. Indeed, China’s public health policy has begun to emphasize “Healthy Aging” and the importance of active lifestyles for older adults; the findings give empirical support to these policy directions. They also suggest that fall prevention campaigns (which traditionally focus on home safety, vision checks, or assistive devices) should incorporate mental health evaluation (52).

Importantly, the urban–rural differences imply that a one-size-fits-all approach may not be optimal. Community centers in urban neighborhoods could partner with mental health professionals to run “mood and mobility” workshops, while rural township clinics might incorporate supervised exercise into their basic public health services for seniors (53, 54). In both settings, raising awareness about the mental health benefits of physical activity is crucial, given that many older adults (especially in China) do not readily recognize or admit to depressive symptoms (55). Our findings support the integration of depression prevention into active aging programs, essentially, promoting exercise is not just a physical health strategy, but a mental health one too.

Several limitations of this study should be acknowledged to contextualize the findings. First, the data are cross-sectional (CHARLS 2020), which constrains our ability to draw causal conclusions. While we tested a mediation model consistent with theory, the temporal ordering between exercise, depression, and falls cannot be definitively established in a single wave. Similarly, an older adult with depression may be less inclined to engage in exercise due to fatigue or lack of motivation, meaning depression could lead to reduced physical activity rather than vice versa. Because of these complexities, our mediation findings should be interpreted as associative rather than strictly causal (56). The second limitation is the reliance on self-reported measures for key variables. Physical exercise frequency/Intensity, depressive symptoms, and falls were all self-reported by respondents, which introduces potential reporting biases. Older adults might over-or under-estimate their exercise and may not accurately recall falls, especially if the falls did not result in injury (57). Depression was measured via a screening scale rather than a clinical diagnosis, which, while common in epidemiological studies, means we captured depressive symptomatology but not necessarily clinically confirmed depression. Third, unmeasured confounding is a concern. We adjusted for a range of demographic and health covariates, but there could be other factors influencing both exercise and falls. For example, there may also be confounders between depression and falls – chronic pain, cognitive impairment, or use of psychoactive medications – that were not captured or fully adjusted in our analysis. Residual confounding might exaggerate or mask parts of the mediation.

Finally, there is the issue of common-method bias since all data came from the same survey source; however, the use of some objectively verifiable outcomes (falls) and the consistency with external studies alleviate this concern somewhat. Despite these limitations, the study provides valuable insights, and we mitigated some concerns (e.g., using validated depression scales, controlling for multiple covariates) to bolster confidence in the findings.

## 5 Conclusion

This study, utilizing data from CHARLS 2020, investigated the relationships among physical activity, depression, and fall risk in older Chinese adults. The findings indicated that engaging in physical exercise effectively reduces depressive symptoms and simultaneously decreases fall risks. Further analysis revealed that depression partially mediated the relationship between physical exercise and fall risk; specifically, regular physical activity reduced depressive symptoms, which in turn contributed to a lower likelihood of experiencing falls. This mediating role of depression highlights the necessity of addressing mental health in combination with physical activity when developing interventions aimed at fall prevention among older adults. While our findings support a mediation pathway, causal claims should be viewed with caution given the data structure.

Notably, the mediating effect of depression was more pronounced in urban areas, possibly due to greater access to exercise resources and increased opportunities for social engagement. Conversely, the effect was weaker among rural residents, likely due to limited availability of exercise facilities and higher baseline depression levels. These results suggest that public health interventions should be context-specific, considering the distinct needs and resource availability in urban and rural settings.

In conclusion, this study emphasizes the importance of integrating physical activity into interventions targeting depression and fall risk reduction among older Chinese adults. Adopting a holistic approach that simultaneously addresses physical and mental health dimensions can significantly enhance the overall well-being of aging populations.

## Data Availability

The original contributions presented in the study are included in the article/supplementary material, further inquiries can be directed to the corresponding author.

## Footnotes

^1^ https://charls.pku.edu.cn/
